# Change in Clinical Risk Factors During Stay and Treatment in a Penitentiary Psychiatric Center

**DOI:** 10.1177/0306624X241282279

**Published:** 2024-10-08

**Authors:** Jennifer Slootmaker, M. Vere van Koppen, Joke M. Harte

**Affiliations:** 1 Custodial Institutions Agency, Ministry of Justice and Security, The Netherlands; 2 Vrije Universiteit Amsterdam, The Netherlands

**Keywords:** clinical risk factors, penitentiary psychiatric center, psychiatric disorders among detainees, forensic treatment, comorbidity, treatment duration

## Abstract

This study examines the changes in clinical risk factors among individuals treated in Dutch Penitentiary Psychiatric Centers (PPCs). Using data from 874 patients with diverse psychiatric disorders, clinical risk factors were assessed at admission and discharge. Slight but significant improvements were observed in most risk factors, particularly psychotic symptoms, over an average stay of approximately 6 months. Patients with psychotic or substance use disorders showed the most improvement, while those with mood, personality, or developmental disorders showed minimal change. The study highlights the positive impact of PPCs’ structured, secure environment and specialized treatment, especially for psychotic disorders. It also underscores the complexity of treating patients with high rates of comorbidity. The findings suggest that the duration of stay does not significantly predict the improvement in most clinical risk factors. Future research should explore specific diagnostic clusters and their impact on treatment outcomes in PPCs.

## Psychiatric Disorders Among Detainees

Worldwide, the prevalence of psychiatric disorders among detainees is high and acknowledged (Diamond et al., 2001; Fazel et al., 2016). It is estimated that one in seven detainees suffers from major depression or psychosis (Fazel & Seewald, 2012), and also substance misuse is a substantial problem among prisoners. In the Netherlands, about 60% of the total prison population has been diagnosed with a mental disorder (DJI, 2024). In specific subpopulations, such as female and remand prisoners, estimates of mental disorder prevalence are even higher (Birmingham, 2004). Detainees with a psychiatric disorder are more likely to exhibit violent behavior in prison (Seidel et al., 2019; Steiner et al., 2014; Walters, 2011) and are at higher risk of both violent and nonviolent recidivism after release, particularly those with comorbid disorders (Fazel et al., 2009; Fazel & Yu, 2011; Grann et al., 2008; Moran et al., 2003; Witt et al., 2013; Yukhnenkok et al., 2023).

## Penitentiary Psychiatric Centers

In the Netherlands, detainees who are incapable of functioning within a regular prison regime due to their mental health and who need specialized psychiatric care can be referred to a Penitentiary Psychiatric Center (PPC). These detainees are in preventive custody or are serving a sentence imposed by the judge. The PPCs can be characterized as psychiatric hospitals located within penitentiary institutions, and detainees are referred to as patients. The quality of this care is equivalent to mental health care in a free society, taking into account the restrictions of the detention environment. Currently, four Dutch PPCs can collectively accommodate 697 patients (7.3% of the total Dutch prison capacity; Van Bekkum et al., 2021). Approximately 1,500 patients are admitted to a PPC annually, with an average stay of 4 months. The main objective of treatment in a PPC is to reduce aggressive and dysfunctional behavior by stabilizing the psychiatric condition and addressing the criminogenic risk factors of a patient. For each patient, a treatment plan is designed upon admission, tailored to their specific psychiatric problems and criminogenic risk factors. A multidisciplinary team regularly evaluates and adjusts this plan (Van Bekkum et al., 2021). As soon as the mental condition is stabilized, the treatment is continued in a regular regime, and for patients whose detention ends during their stay in a PPC, aftercare is organized. The aim of treatment in a PPC is for the detainee to return safely to society.

## Clinical Risk Factors: Change, Psychopathology, and Duration of Treatment

In studies on forensic populations, risk assessment instruments are used to describe the patients’ characteristics and monitor individual treatment progress in the forensic setting. In the PPCs, the clinical risk factors of the Historical Clinical Future-Revised (HKT-R; Spreen et al., 2014) are used to monitor PPC patients during their stay, whereas some other studies (e.g., De Vries Robbé et al., 2014) used the Historical Clinical Risk Management-20 (HCR-20; Webster et al., 1997). Clinical risk factors measured with risk assessment instruments are so-called dynamic factors that are susceptible to change and are supposed to be related to the risk of recidivism (Bonta & Andrews, 2007; De Vries Robbé et al., 2014; Eisenberg et al., 2019).

Previous studies on the effect of forensic treatment on clinical risk factor scores focused on the effect of forensic treatment imposed by the court and yielded mixed results. While some studies did not find an effect on the clinical scale of risk factors (Longdon et al., 2018; Schuringa et al., 2019; Van der Vreeken et al., 2018; Verschueren et al., 2023), most studies found significant but small improvement on clinical risk factors over time (e.g., Janković et al., 2021; Olsson et al., 2013; Richter et al., 2018). De Vries Robbé et al. (2014), for example, explored a sample of 108 discharged, high-risk forensic psychiatric male patients, predominantly diagnosed with a personality disorder. On average, they were treated for 5.7 years in a long-term forensic inpatient program. They found a decrease in pre- to post-treatment scores on the clinical risk factor scale for violent recidivism and showed that improvement is also related to abstention from violence for short- as well as long-term follow-up. Van der Linde et al. (2020) investigated the changes in dynamic risk factors during forensic treatment while taking into account the patient’s age, treatment duration, and whether patients were first-time offenders or recidivists. Based on a sample of 317 patients, they found a decrease of risk factors over time, though no differences were found between first-time offenders and recidivists and between different age groups.

It should be noted that the psychopathology of samples used in the different studies varied, which might explain why some studies did not find an effect of forensic treatment while others did. The few studies that examined the relationship between psychopathology and change in clinical risk factors as a response to forensic treatment strengthen this explanation. Janković et al. (2021), for example, investigated whether changes in clinical risk factors during treatment differ between patients *with* and *without* substance use disorder, psychotic disorder, and cluster B personality disorder. Patients with psychotic disorders showed more improvement on the clinical scale during the first phase of their stay in the forensic psychiatric center than patients without these disorders. The rapid progress might partly be explained by initial levels of risk factors, which were substantially higher for patients with a psychotic disorder than for patients without a psychotic disorder. Patients without a psychotic disorder showed relatively low scores at the first measurement. No differences were found in change on the clinical subscale between patients with and without a substance use disorder and between patients with and without a cluster B personality disorder.

Although the range in duration in forensic psychiatric treatment is extensive in most samples, only a few previous studies examined the effect of length of treatment or stay in a forensic hospital on change in risk factors for recidivism. Van der Linde et al. (2020) included 179 patients in examining the effect of the length of stay. On average, they were treated for a period of 5.9 years. No differences were found between patients with a treatment duration of less than 5 years, between 5 and 7 years, and longer than 8 years in their change in dynamic risk factors during treatment. Van der Vreeken et al. (2018) compared patients with their first assessment within 12 months after admission to patients who had been in treatment for a longer period and did not find a difference in progress on problem behavior between both groups. Some study findings suggest that improvement in risk factors mainly occurs during the first phase of treatment (Janković et al., 2021; Richter et al., 2018), more specifically during the first 14 months of treatment (Nijman et al., 2004). If this is the case, significant differences between a stay of 14 months and stays longer than that are not expected. However, in another study, reductions in some clinical risk factors were found after 9 months of treatment; all but two risk items had changed after a mean of 43 months of treatment, indicating that a more extended treatment results in more progress (Olsson et al., 2013).

## Current Study

Previous research on the changeability of clinical risk factors produced varying results, which can be explained by the variation in study design, patient population, sample, and duration of treatment. Samples used in previous studies are often limited in size, and those studies fail to consider the heterogeneity of the sample, for example, with regard to psychopathology or duration of treatment. It was already recommended to differentiate between patients with different disorders when studying treatment progress (see, e.g., De Jonge et al., 2009). Earlier studies on change in risk factors during treatment in a psychiatric forensic setting often used sum or mean scores (of subscales) of risk assessment instruments instead of exploring the possible change of each unique factor separately. An exception is the study by Verschueren et al. (2023), in which no change was found for the clinical scale as a whole, but an improvement after treatment was found for the problem insight of patients. Although the range in duration of forensic psychiatric treatment is extensive in most samples, few studies thus far that looked into pre- and posttreatment risk factor scores did take the duration of treatment into account.

Using a large sample and repeated measurements of clinical risk factors, this study aims to provide more insight into the effect of treatment in a PPC and into which factors of forensic treatment do or do not contribute to the improvement on clinical risk factors. Patients with different psychiatric disorders may exhibit dysfunctional behavior in different areas or may vary in their degree of susceptibility to changes in risk factors. Also, the duration of treatment might be related to improvement. The study is guided by the following research questions:

*Q1: Do scores on clinical risk factors change during the stay and treatment at a Penitentiary Psychiatric Center?*
*Q2a: Do scores on clinical risk factors change during the stay and treatment at a Penitentiary Psychiatric Center for patients diagnosed with a developmental disorder, psychotic disorder, mood disorder, substance use disorder, or personality disorder?*
*Q2b: How are these changes affected by comorbidity?*
*Q3: Is the change in clinical risk factors influenced by the time between the two measurements (and thus the length of stay and treatment in a PPC)?*

The total group of PPC patients is expected to improve on many if not all, clinical risk factors over the course of their stay (Q1). A difference is expected in the effect of stay and treatment for patients diagnosed with different disorder groups, as reflected by improvement in more clinical risk factors for some diagnostic groups than for others. As admission to a PPC can be regarded as a crisis intervention, it is expected that patients with a psychotic disorder benefit more from the structure and treatment, quite often with antipsychotic medication, than patients with other diagnoses. On the other hand, patients with more persistent disorders are expected to show less improvement as a result of treatment and stay in a PPC (Q2a). Furthermore, it is likely that when comorbidity is considered, effects that are found earlier disappear for some diagnostic groups as they appear to be spurious effects. This study defines comorbidity as the co-occurrence of two or more disorder groups (Q2b). The length of stay and the time between T1 and T2 varies between patients. Patients with a longer stay are expected to benefit more from the structure, stability, and treatment during their stay than patients with a shorter stay and, therefore, show more improvement on the risk scores (Q3).

## Methodology

### Sample

Patient data for all individuals admitted to one of the PPCs in the Netherlands are systematically collected and compiled into a national database. Between January 1, 2019, and April 1, 2022, 4,795 patients were admitted to the PPCs. In the case of multiple admissions of the same patient in this period, only data from the most recent admittance were included, resulting in a sample of 2,943 unique patients. Criminogenic risk factors were scored 7 weeks after admittance (T1), annually in case of a long stay, and upon discharge under the condition that the period between the previous measurement (admission or annual measurement) and discharge is at least 6 weeks. The last measurement was used as T2. Because patients only have a T1 and T2 when they stay at least 13 weeks in a PPC, all patients with a shorter stay are excluded from the study. Of the remaining 1,144 patients, 270 had missing measurements, resulting in a final study sample of 874 patients. Comparisons between both groups were made to determine whether the patients with missing measurements (*n* = 270) differed from the resulting sample (*n* = 874). Women appeared to be underrepresented in the group with missing scores ( $\chi^{2}$ = 21.58, *p* < .001, *phi* = −.137). No difference was found for age (*p* = .806) and length of stay (*p* = .766).

### Measures

The 15 clinical items in the HKT-R used in this study are (1) lack of insight into own problems, (2) psychotic symptoms, (3) addictive behavior, (4) impulsivity, (5) antisocial behavior, (6) hostility, (7) lack of social skills, (8) lack of self-reliance, (9) non-cooperation with treatment, (10) lack of responsibility for the offense, (11) lack of coping skills, (12) violation of terms and agreements, (13) lack of employment skills, and (14) negative network. In the PPCs, the risk factor (15) sexual preoccupation, which was dropped after a revision of the HKT, is also scored. All items are scored on a 5-point scale (from 0 to 4), with higher scores indicating more dysfunctional behavior. The internal consistency and interrater reliability of the Clinical domain in the original HKT-R were found to be good (α = .86; ICC = .85; Spreen et al., 2014).

For each patient, a DSM-V diagnosis results from a consensus diagnosis based on independent primary interviews with a psychiatrist and a psychologist. Subsequently, DSM diagnoses are categorized into five broader categories for the purpose of this study. The length of stay and treatment is approximated by the number of weeks between T1, administered 7 weeks after admission, and T2, administered at discharge.

### Analyses

For all clinical risk factors, descriptives on T1 and T2 and the difference score (T1-T2) are computed and tested for significance (Q1). The same procedure was used for diagnostic groups separately (Q2a). Multiple linear regressions are used for each clinical risk factor to disentangle the comorbid effects of different diagnostic groups (Q2b) and to determine the effect of length of stay (Q3) on the progression of individual risk factors.

## Results

### Characteristics of the Sample

The sample comprises 815 men (93%) and 59 women (7%). The mean age at admission is 36.5 years (*SD* = 12.2, range 18–85 years), and patients stayed 32.8 weeks on average (*SD* = 20.1, range 13–136). Most patients had at least one prior criminal conviction (80.5%), and one out of five (18.5%) had been admitted to a PPC before. The mean age at the first registered offense was 21.7 years (*SD* = 9.65, range 10–78), and for violent offenses, it was 23.7 years (*SD* = 10.37, range 10–78). Index offenses, that is, offenses leading to the current detention, are categorized according to the BooG system (Brand, 2005). Index offenses are murder (8.7%), arson (8.9%), manslaughter (14.4%), sexual offense with an adult victim (5.3%) or juvenile victim (1.9%), aggravated assault (11.0%), property offense involving violence (10.6%), moderate violent offense (25.2%), profit property offense (10.5%), property offense such as vandalism of goods (1.3%), drug-related offense (1.9%), and a minor offense such as a traffic offense (0.1%). All patients are diagnosed with at least one psychopathological disorder, with 62.7% of the patients diagnosed with two or more disorders and 18.6% diagnosed with four or more disorders. After categorizing the diagnoses, the most prevalent diagnostic groups were psychotic disorders (58.7%), substance-related disorders (43.4%), developmental disorders (22.7%), personality disorders (21.5%), and mood disorders (11.0%). Based on these broader disorder categories, 37.2% have two comorbid disorders, 11.5% are diagnosed with three comorbid disorders and 0.7% have four comorbid disorders.

### Clinical Risk Factors and Change

For the total sample, Table 1 shows scores on clinical risk factors at admission (T1), discharge (T2), and change scores (T1–T2). Results indicate a significant (*p* < .05) but slight improvement throughout the stay in a PPC on all risk factors except for *addictive behavior* and *negative network*. The most significant change was found for *psychotic symptoms* ( ΔM *=* 0.38, Cohen’s *d* = 0.33).

**Table 1.** Clinical Risk Factors at T1 and T2.

Risk factors (range 0–4)	*n*	T1	T2	T1–T2	*d*
		*M* (*SD*)	*M* (*SD*)	ΔM (*SD*)	
Lack of insight into own problems	860	2.37 (1.26)	2.17 (1.23)	0.20 (1.23)***	0.16
Psychotic symptoms	869	1.08 (1.21)	0.70 (1.01)	0.38 (1.18)***	0.33
Addictive behavior	865	0.31 (0.65)	0.30 (0.63)	0.01 (0.70)	0.02
Impulsivity	860	1.37 (1.29)	1.18 (1.19)	0.19 (1.29)***	0.14
Antisocial behavior	862	0.89 (1.05)	0.76 (1.02)	0.13 (1.04)***	0.13
Hostility	866	1.02 (1.00)	0.85 (0.95)	0.17 (0.04)***	0.16
Lack of social skills	865	1.48 (0.97)	1.36 (0.93)	0.12 (1.00)***	0.12
Lack of self-reliance	866	1.24 (1.19)	1.15 (1.18)	0.10 (1.11)*	0.09
Noncooperation with treatment	851	1.63 (1.24)	1.52 (1.19)	0.12 (1.23)**	0.09
Lack of responsibility for offense	728	2.10 (1.22)	1.90 (1.28)	0.20 (1.17)***	0.17
Lack of coping skills	859	2.03 (1.04)	1.88 (1.04)	0.15 (1.13)***	0.13
Violation of terms and agreements	862	0.79 (1.13)	0.69 (1.05)	0.10 (1.22)*	0.08
Lack of employment skills	792	1.11 (1.36)	0.95 (1.23)	0.16 (1.36)***	0.12
Negative network	746	0.91 (0.93)	0.84 (0.95)	0.07 (0.98)	0.07
Sexual preoccupation	568	0.24 (0.63)	0.15 (0.49)	0.09 (0.62)***	0.15

*Note. d* = Cohen’s *d*.

* *p* < .05. **p < .01. ***p < .001.

### Clinical Risk Factors and Psychopathology

Change scores for all risk factors and five diagnostic groups separately are shown in Table 2. It should be noted that comorbidity is high, and patients may belong to multiple diagnostic groups. Patients diagnosed with a psychotic disorder and patients diagnosed with a substance use disorder show improvement on most of the risk factors during their stay. Improvement was most substantial for *psychotic symptoms* (*d* = 0.38 and *d* = 0.34, respectively). Limited progress was found for patients affected by a developmental, personality disorder, or mood disorder. Patients with a personality disorder were found to have improved scores on *Psychotic symptoms* only. The group of patients affected by a mood disorder only appeared to improve scores on *Violation of terms and agreements* and was also the only subgroup to show improvement on this risk factor. No improvement of scores was found for patients affected by a developmental disorder. The most significant improvement was seen in psychotic symptoms when results were examined by risk factor, with substantial improvement and small to medium effect sizes for all subgroups; no subgroup improved on addictive behavior during their stay at a PPC.

**Table 2.** Clinical Risk Factors at T1 and T2, Distinguished by Psychiatric Disorder.

Risk factors (range 0–4)	Developmental disorders (*n* *=* 198)		Psychotic disorders (*n* *=* 513)		Mood disorders (*n* *=* 96)		Substance use disorders (*n* *=* 379)		Personality disorders (*n* *=* 188)	
	ΔM	*d*	ΔM	*d*	ΔM	*d*	ΔM	*d*	ΔM	*d*
Lack of insight into own problems	0.15	0.13	0.25***	0.20	0.09	0.07	0.27***	0.21	0.08	0.06
Psychotic symptoms	0.24**	0.20	0.49***	0.38	0.28*	0.25	0.39***	0.34	0.33***	0.33
Addictive behavior	0.02	0.03	0.01	0.01	0.05	0.11	0.02	0.03	0.03	0.03
Impulsivity	0.12	0.09	0.23***	0.18	0.42**	0.33	0.14*	0.11	0.08	0.06
Antisocial behavior	0.10	0.09	0.20***	0.20	0.11	0.11	0.20***	0.19	0.16	0.13
Hostility	0.07	0.06	0.20***	0.20	0.26**	0.28	0.20**	0.17	0.14*	0.15
Lack of social skills	0.11	0.11	0.14***	0.15	0.25**	0.28	0.11*	0.11	0.09	0.09
Lack of self-reliance	0.08	0.07	0.14**	0.12	0.10	0.09	0.01	0.01	0.05	0.05
Noncooperation with treatment	0.20*	0.17	0.15**	0.13	0.19	0.15	0.13*	0.11	−0.02	−0.02
Lack of responsibility for offense	0.19	0.15	0.21***	0.18	0.21	0.17	0.18*	0.14	0.26**	0.21
Lack of coping skills	0.02	0.02	0.19***	0.17	0.24	0.21	0.19**	0.15	0.03	0.02
Violation of terms and agreements	−0.07	−0.06	0.13*	0.11	0.33***	0.36	0.03	0.03	0.05	0.04
Lack of employment skills	0.13	0.09	0.23***	0.17	0.37**	0.30	0.14*	0.11	0.07	0.05
Negative network	0.03	0.03	0.05	0.05	0.12	0.15	0.16**	0.16	0.12	0.11
Sexual preoccupation	0.04	0.06	0.12***	0.18	0.07	0.12	0.09*	0.15	0.09	0.13

*Note. ΔM* = change score from T1 to T2; *d* = Cohens’s *d*.

* *p* < .05. **p < .01. ***p < .001.

### Clinical Risk Factors and Duration of Treatment

Multiple linear regression analyses were performed to (1) further disentangle the comorbid effects of different diagnostic groups and (2) to determine the relationship between the diagnostic group and each risk factor separately while considering the possibility that the patient is also diagnosed in another disorder group. The fact that patients diagnosed with substance use disorders show improvement on many risk factors may be explained by the fact that many of them have, for example, a comorbid disorder. In short, these analyses give insight into how comorbidity influences the previous results. In addition, length of stay is included in the analyses as an explanatory variable to examine the effect of length of stay on progression on individual risk factors.

As shown in Table 3, only 4 of 14 clinical risk factors could be successfully predicted by one or more explanatory variables. While taking into account diagnoses of other disorder group(s), patients with a *psychotic disorder* (β = .112, *p* < .01) were expected to improve on *psychotic symptoms* over the course of their stay. Scores on a*ntisocial behavior* were expected to improve for patients with a psychotic disorder (β = .087, *p* < .05) and for patients with a substance use disorder (β = .069, *p* < .05). With regard to scores on Violation of terms and agreements, it was expected that patients with a developmental disorder show less progress between T1 and T2 time than patients without a developmental disorder (β = −.079, *p* < .05). Scores on *negative network* were expected to improve when a *substance use disorder* was diagnosed (β = .086, *p* < .05). The time between T1 and T2, reflecting length of stay, is only related to a small but significant improvement on *psychotic symptoms* (β = .083, *p* < .05). The length of stay is not related to a change in any of the other clinical risk factors.

**Table 3.** Prediction of Change in Clinical Risk Factors by Diagnostic Groups and Time Between Measurements.

Models	Dependent variable (risk factor)	Explanatory variables	*B*	*SE*	95% CI	β	*R* ^2^	*F*
Model 1	Psychotic symptoms						.019	8.379***
		Psychotic disorders	0.268	0.081	[0.110, 0.426]	.112**		
		Time between T1 and T2	0.005	0.002	[0.001, 0.009]	.083*		
Model 2	Antisocial behavior						.011	4.701**
		Psychotic disorders	0.183	0.072	[0.041, 0.325]	0.087*		
		Substance use disorders	0.145	0.072	[0.004, 0.286]	0.069*		
Model 3	Violation of terms and agreements						.006	5.449*
		Developmental disorders	−0.232	0.099	[−0.426, −0.037]	−.079*		
Model 4	Negative network						.007	5.544*
		Substance use disorders	0.170	0.072	[0.028, 0.311]	.086*		

*Note. SE* = standard error; CI = confidence interval; β = standardized coefficient.

* *p* < .05. **p < .01. ***p < .001.

## Conclusions and Discussion

This study aimed to explore possible reduction in clinical risk factor scores during the stay and treatment at a Penitentiary Psychiatric Center (PPC). Overall, a significant improvement in risk factors throughout the stay was found at the group level, indicating a positive effect of the structure, stability, and treatment in a forensic setting on the behaviors of its patients. Effect sizes are small, though they reflect a change in risk factors in a relatively short period (average stay equals half a year). Most substantial changes were found in *psychotic symptoms*.

Although these findings resemble those of previous studies examining the effects of forensic treatment on clinical risk factors (e.g., De Vries Robbé et al., 2014; Janković et al., 2021; Olsson et al., 2013; Van der Linde et al., 2020; Richter et al., 2018), they still are surprising given the fact that measurements are based on staff observations of dysfunctional behavior, which are less sensitive to change than, for example, self-report measurements. Furthermore, the sample had an average period between pre- and post-treatment risk scores of 25.9 weeks (*SD* = 20.2, median = 20 weeks), much shorter than samples in most other studies. Previous studies mainly focused on long-term forensic treatment imposed by the court with longer follow-ups. However, treatment in a PPC can be regarded as a crisis intervention. As a result, the PPC population, with a majority of patients suffering from a psychotic disorder often combined with at least one other diagnosis, differs from those in the other studies. It has previously been suggested that the reduction of risk factors is a slow process (Hildebrand & De Ruiter, 2012) and that a two-year treatment period is too short to detect improvement in a high-security patient population (Verschueren et al., 2023). Furthermore, the HKT-R has been criticized for its lack of sensitivity to detect change, particularly in the first months or years of treatment (Longdon et al., 2018; Nitschke et al., 2020; Olsson et al., 2013; Richter et al., 2018; Schuringa et al., 2019; Verschueren et al., 2023). The current study shows that reduction in risk scores is also possible over shorter treatment periods and using the HKT-R, at least for patients diagnosed with a psychotic disorder.

When different diagnostic groups are distinguished, patients diagnosed with a psychotic disorder are the largest group (58.7%), often combined with at least one other diagnosis. Patients with a psychotic disorder are not only the largest group in the PPC, but they also appear to benefit the most from their stay in a PPC, as reflected by their improvement on the clinical risk factors. PPCs are designed to offer a structured and secure environment that is particularly beneficial for patients affected by a psychotic disorder. This environment can help alleviate stress and anxiety, which are known to exacerbate symptoms of psychotic disorders (Mueser et al., 2003). Secondly, the beneficial effects for psychotic patients in the PPC can be explained by the fact that they may have received more intensive and specialized treatment, including medication management or compulsory medication, than patients with other diagnoses. The effectiveness of antipsychotic medication in reducing psychotic symptoms and improving overall functioning in patients with schizophrenia and related disorders has been well-established in previous research (Haddad & Correll, 2018; Leucht et al., 2013). Reduction of psychotic symptoms may be achieved by participation in therapeutic interventions such as cognitive-behavioral therapy, social skills training, and other interventions offered in the PPC, leading to further improvements on risk factors (Barrowclough et al., 2011; Wykes et al., 2008).

The second-largest group, patients diagnosed with substance use disorder, also show improvement on many of the risk factors, but this could partly be explained by the overlap with a psychotic disorder diagnosis, that is, 142 out of 379 (37.5%) patients diagnosed with a substance use disorder are also diagnosed with a psychotic disorder. This assumption seems to be validated by analyses taking into account this comorbidity, showing that only for a decrease in antisocial behavior *both* a psychotic disorder diagnosis and substance use disorder are predictive.

Patients diagnosed with a mood disorder, personality disorder, or developmental disorder showed improvement on none or only a single risk factor. This may be due to the complexity of these disorders and the possibility that these disorders are more resistant to treatment. Also, comorbid conditions can complicate treatment and make it more difficult to achieve positive outcomes (McHugh & Weiss, 2019). Patients with developmental disorders, for example, often experience chronic and persistent behavioral and functional impairments due to their strong biological basis (Gillberg & Billstedt, 2000). These disorders are usually diagnosed early in life and persist into adulthood. However, interventions that target developmental disabilities may not necessarily address the associated behavioral and functional impairments (Mazurek & Kanne, 2010).

This study was able to show the effect of a stay in a PPC on a wide range of clinical risk factors that are supposed to be related to the risk of recidivism. It also furthers our understanding of which patients benefit most from treatment in a PPC. At the same time, this study underlined the complexities of the high rates of comorbid disorders in a PPC and how these interfere with each other. Future research should aim to identify specific clusters of disorders and explore the direct effects of these diagnostic clusters on the change in behavior during stay and treatment in a PPC. No effect was found for the length of stay on all but one clinical risk factor. This finding suggests that shorter and extended stays can be related to improvement on most clinical risk factors. Still, it might as well be explained by methodological limitations, that is, the range and distribution of length of stay in this study were not significant or varied enough to find an effect of length of stay. Over 60% of the patients stay in a PPC shorter than 13 weeks and are excluded from the current study because their clinical risk factors were not measured twice. Therefore, the findings of the current study only apply to those with a relatively long stay, which might imply that their dysfunctional behavior is more severe than those with a shorter stay.

While the study demonstrated the improvement of risk factors during a stay in a PPC, it did not identify specific aspects of treatment in the PPC that contributed to these changes. Future research should aim to provide a more comprehensive understanding of treatment progression beyond length of stay and diagnosis by assessing multiple predictors and treatment efforts. For instance, exploring variables such as pharmacotherapy or therapeutic interventions can provide insight into the aspects of a stay in a PPC that contribute to observed changes in dysfunctional behavior.

The findings of this study have important implications for the treatment of detained individuals with psychiatric disorders. Specifically, the study highlights the potential effectiveness of specialized psychiatric care, such as that provided in PPC facilities, in reducing dysfunctional behavior among forensic patients, particularly those diagnosed with psychotic disorders.
